# Complicated appendicitis: value of inflammatory markers based on EAES 2015 guidelines

**DOI:** 10.1007/s00464-025-12038-z

**Published:** 2025-08-18

**Authors:** Maximilian Dölling, Michael Klös, Jonas Pachmann, Jessica Stockheim, Mirhasan Rahimli, Sara Al-Madhi, Ulf D. Kahlert, Aristotelis Perrakis, Martin Herrmann, Roland S. Croner, Mihailo Andric

**Affiliations:** 1 Faculty of Medicine, University Clinic for General-, Visceral-, Vascular- and Transplantation Surgery, Otto-Von-Guericke-University, Leipziger Str. 44, 39120 Magdeburg, Germany; 2 Molecular and Experimental Surgery, Department of General-, Visceral-, Vascular and Transplant Surgery, Faculty of Medicine and University Hospital Magdeburg, Otto-Von-Guericke University, 39120 Magdeburg, Germany; 3 Iatriko Medical Center, Department of General, Minimally-Invasive Surgery and Surgical Oncology, Hepatopancreatobiliary and Advanced Colorectal Surgery, Athens, Greece

**Keywords:** Phlegmonous appendicitis, Complicated acute appendicitis, C-reactive protein, Inflammatory biomarkers, Postoperative morbidity, EAES classification

## Abstract

**Background:**

The clinical course of phlegmonous appendicitis (PHL) appears less severe than other complicated forms, which led to the discussion if PHL should be classified as complicated acute appendicitis (CAA). According to the guidelines of the European Association for Endoscopic Surgery (EAES) from 2015, PHL is classified as a CAA, which requires usually surgery. Therefore, this study aimed to evaluate the relevance of classifying PHL as CAA, with a focus on inflammatory markers, postoperative complications, and hospital length of stay.

**Methods:**

We conducted a retrospective single-center study including 559 adult patients who underwent appendectomy between 2016 and 2020. Intraoperative classification followed the EAES 2015 guidelines. Preoperative C-reactive protein (CRP) and leucocyte counts were analyzed with respect to disease severity, postoperative complications, and length of hospital stay.

**Results:**

Complicated appendicitis was diagnosed in 62.5% of patients, with phlegmonous appendicitis accounting for 30.8%. CRP levels were significantly higher in CAA than uncomplicated acute appendicitis (UAA; Median 48.4 vs. 8.8 mg/L; *p* < 0.001) and increased progressively with disease severity. CRP showed good diagnostic performance in distinguishing CAA from UAA (AUC: 0.76), whereas leucocyte count demonstrated limited diagnostic utility (AUC: 0.57). With suspected appendicitis at a CRP threshold of 52.5 mg/L, at least PHL could be expected (49% sensitivity, 95% specificity, AUC 0.72). The risk of postoperative complications was higher in all CAA subtypes, including PHL (adjusted OR: 2.3; *p* = 0.015). Elevated preoperative CRP levels were associated with both complications (*p* < 0.001) and prolonged hospital stay (IRR: 1.21 for PHL; *p* < 0.001). Leucocyte counts were neither predictive of complications nor hospital duration.

**Conclusion:**

Phlegmonous appendicitis shows a distinct clinical profile with moderate CRP elevation, a 2.3-fold increased risk of postoperative complications, and a 21% increase in hospital stay. These findings support its classification under EAES 2015 guidelines as complicated appendicitis.

Acute appendicitis (AA) is one of the most common abdominal surgical emergencies, with a pooled annual incidence of 100–151 cases per 100,000 individuals in western countries [1]. The lifetime risk of developing AA is 8.6% for males and 6.7% for females, with a peak incidence in males aged 10–14 years and females aged 15–19 years [2, 3]. The socioeconomic impact of AA is significant, with appendicitis-related hospital admissions contributing to increasing healthcare costs [4].

Surgical appendectomy is considered the gold standard treatment, though efforts were made to provide alternative therapies for less severe cases of acute appendicitis [5]. Thus, large clinical trials explored non-operative treatment (NOT) using antibiotics as an alternative therapy for cases considered as “uncomplicated” acute appendicitis [6, 7]. However, there is an ongoing debate regarding the long-term effectiveness, recurrence rates, and patient selection criteria [8, 9]. Recent meta-analyses suggest that while NOT can be successful in selected patients with non-severe clinical presentation and without risk factors such as the presence of appendicoliths, recurrence rates remain a concern, with up to 40% requiring subsequent appendectomy within a year and almost 49% within 4 years [10–12]. Therefore, accurate identification of patients suitable for NOT remains clinical challenging and a major focus of research.

Also, a major challenge in the management of AA lies in the lack of a universally standardized classification system for distinguishing uncomplicated (UAA) from complicated AA (CAA) that assist clinical decision-making. As a consequence, international conferences tried to establish uniform diagnostic and treatment criteria [13, 14]. According to the guidelines of the European Association of Endoscopic Surgery (EAES) in 2015, UAA is characterized by simple inflammation of the appendix without surrounding reaction, whereas CAA is defined by findings such as free abdominal fluid, surrounding phlegmon, intraabdominal abscesses, gangrene, or perforation [15]. Additionally, the presence of appendicoliths has been recognized as a risk factor for perforation [16]. The presence of appendicoliths or any of these criteria leads to the decision of appendectomy [17].

The diagnosis of AA is based on patient history, clinical presentation, physical examination, and imaging such as abdominal ultrasound and CT scans [18–20]. Furthermore, laboratory markers, including C-reactive protein (CRP), leucocyte count, and differential blood counts, are routinely used to identify intraabdominal inflammation [21]. However, despite advancements in imaging, accurately classifying AA remains difficult [22, 23]. To improve diagnostic precision, various laboratory markers—such as CRP-to-lymphocyte ratio, leucocyte-to-lymphocyte ratio, procalcitonin, hyperbilirubinemia, and bilirubin-to-leucocyte ratio—have been investigated [24–27]. Additionally, scoring systems like the Alvarado score and the Appendicitis Inflammatory Response (AIR) score integrate patient characteristics and clinical findings to enhance diagnostic accuracy [28, 29].

The diagnostic utility of CRP and leucocyte count in predicting advanced forms of AA have been investigated in previous studies, typically mainly counting gangrenous or perforated appendicitis to CAA [29, 30]. However, inconsistencies in subgroup classification and the lack of integration with updated EAES criteria—particularly the classification of phlegmonous appendicitis as CAA—have limited their applicability [15]. Therefore, the aim of this study was to investigate the diagnostic and prognostic value of C-reactive protein (CRP) and leucocyte count in acute appendicitis, using the 2015 classification framework of the European Association for Endoscopic Surgery (EAES). We hypothesized that CRP is superior to leucocyte count in distinguishing uncomplicated from complicated appendicitis, particularly when phlegmonous inflammation is classified as a complicated form. Furthermore, we aimed to evaluate whether phlegmonous appendicitis in the framework of EAES as well as inflammatory markers is associated with postoperative complications and prolonged hospital stay.

## Patients and methods

### Study design

This study is a retrospective single-center study conducted at the University Clinic for General-, Visceral-, Vascular- and Transplantation Surgery, Otto-von-Guericke University Magdeburg, a tertiary academic teaching hospital in Germany. The study was designed and carried out in compliance with the principles of "good clinical practice" and the Declaration of Helsinki. It was not prospectively registered as it was retrospective in nature. The protocol was approved by an institutional review board (EA 173/22). All participants received detailed patient information and provided written informed consent. Also, this study was conducted in accordance with STROBE/STROCSS 2021 reporting guidelines. The authors confirm the completeness and accuracy of the data.

### Patients

From 2016 to 2020, all patients over the age of 18 who underwent surgical appendectomy, had a clinical diagnosis of acute appendicitis, and provided written informed consent were included in the study. Patients diagnosed with appendicitis but treated conservatively were excluded from the study.

No formal sample size calculation was performed due to retrospective nature. All available patient data were included without imputation for missing values. To minimize selection bias, all consecutive adult patients who underwent appendectomy for suspected acute appendicitis during the study period were included.

### Institutional management protocol

At our institution, intravenous antibiotics are routinely initiated in the emergency department upon clinical diagnosis of acute appendicitis. All patients included in this study underwent laparoscopic appendectomy. In cases of perforated appendicitis with abscess formation, surgical treatment is preferred; percutaneous drainage is considered only for abscesses exceeding 5 cm. Surgical drains are selectively placed in perforated cases with diffuse peritonitis or large fluid collections. Imaging is routinely performed preoperatively, with abdominal ultrasound as the first-line modality and CT scans used in cases of diagnostic uncertainty. Imaging is interpreted by certified radiologists in daytime and resident radiologists overnight. Time-to-operation typically ranges from 4 to 8 h after ED admission, depending on OR capacity and clinical urgency. Perforated cases are prioritized for earlier surgical intervention.

### Exposures

Preoperative C-reactive protein (CRP) levels [mg/L] and leucocyte counts [Gpt/L] were defined as the primary exposure variables. These values were collected at the time of admission, prior to antibiotic administration or surgery.

### Outcomes

The primary outcome was the intraoperative classification of acute appendicitis according to the EAES 2015 guidelines. Secondary outcomes included 30-day postoperative morbidity (complications) and length of hospital stay [days]. Hospitalization time was defined as the duration from admission to the emergency department until discharge.

### Definitions

According to the 2015 guidelines of the European Association of Endoscopic Surgery (EAES), we classified Uncomplicated Acute Appendicitis (UAA) as catarrhal appendicitis, and Complicated Acute Appendicitis (CAA) as phlegmonous-, gangrenous-, or perforated appendicitis, including perityphlitic abscess, based on intraoperative findings reported by trained surgeons. Phlegmonous appendicitis (PHL) is defined as an inflamed vermiform appendix without gangrene but with local periappendicular inflammation and free abdominal fluid. Gangrenous appendicitis (GAN) is characterized by phlegmonous appendicitis with gangrenous changes in the vermiform appendix. An abscess (ABS) is defined as an inflamed vermiform appendix without obvious acute perforation but with a perityphlitic puss collection. Perforated appendicitis (PER) is defined as an inflamed vermiform appendix with an open or covered perforation, accompanied by localized or spread peritonitis.

Postoperative morbidity was defined according to the Clavien–Dindo classification. Any deviation from the normal postoperative course requiring pharmacological treatment, surgical, endoscopic or radiological intervention, or intensive care admission was considered a complication (Clavien–Dindo grade ≥ II).

### Statistical analysis

Data collection was performed using Microsoft Excel (v16.79.1, Microsoft, Redmond, WA, USA). Statistical analysis was performed using STATA (v17.0, StataCorp, College Station, TX, USA). Descriptive statistics were calculated for baseline characteristics of the study population, including median values with interquartile ranges (IQR) for continuous variables and frequencies with percentages for categorical variables. To compare differences between groups, non-parametric tests were used due to the non-normal distribution of the data. The Wilcoxon rank-sum test was employed for two-group comparisons, while the Kruskal–Wallis test was applied for comparisons across more than two groups. For post hoc pairwise comparisons following the Kruskal–Wallis test, Bonferroni correction or Dunn’s test was applied. Receiver operating characteristic (ROC) curves were applied. Optimal cutoff values were determined by using Youden index. The association between continuous variables, such as C-reactive protein concentration and leucocyte counts, with the length of hospitalization was assessed using Spearman’s correlation. For the analysis of postoperative complications, a Chi-squared test was used to assess differences between uncomplicated and complicated appendicitis groups. Also, logistic regression was applied to estimate odds ratios (ORs) for postoperative complications between appendicitis subtypes. The impact of appendicitis classification on the length of hospital stay was assessed by a negative binomial regression model to account for overdispersion in hospitalization data and expressed as Incidence Rate Ratios (IRRs) with 95% confidence intervals (CIs). Statistical significance was set at *p* < 0.05 two-sided for all tests. Data visualization, including bar graphs, box plots, and ROC curves, was performed using GraphPad Prism (v10.4.1, GraphPad Software, Inc, Boston, MA, USA).

## Results

### Patient characteristics and clinical findings

A total of 559 patients were included in the study (Table 1), with a median age of 38 years (IQR: 26–58) and a median BMI of 25.6 kg/m^2^ (IQR: 22.9–29.2). The cohort consisted of 301 (53.8%) male and 258 (46.2%) female patients. The median BMI was 25.6 (IQR: 22.9–29.2) with the majority of patients (42.8%) having a BMI within the normal range (18.5–24.9 kg/m^2^), while 34.4% were classified as overweight (25.0–29.9 kg/m^2^), and 20.8% were obese (≥ 30.0 kg/m^2^). Diabetes was present in 7.2% of patients. Regarding the American Society of Anesthesiologists (ASA) classification, most patients had an ASA score of I (33.9%) or II (54.1%), with 12.0% classified as ASA ≥ III. Inflammatory markers showed a median leucocyte count of 14.1 Gpt/L (IQR: 11.2–17.2) and a median C-reactive protein (CRP) level of 23.4 mg/L (IQR: 5.2–72.4). Complicated appendicitis according to guidelines of the European Association of Endoscopic Surgery from 2015 (EAES) was observed in 349 patients (62.5%), including phlegmonous (30.8%), gangrenous (10.2%), abscess-forming (6.3%), and perforated (15.0%) appendicitis. Uncomplicated appendicitis was diagnosed in 209 patients (37.5%). Postoperative complications occurred in 60 patients (10.7%), with 6.3% classified as minor complications (Clavien-Dindo < IIIA) and 4.4% as major complications (Clavien–Dindo ≥ IIIA).

**Table 1:**
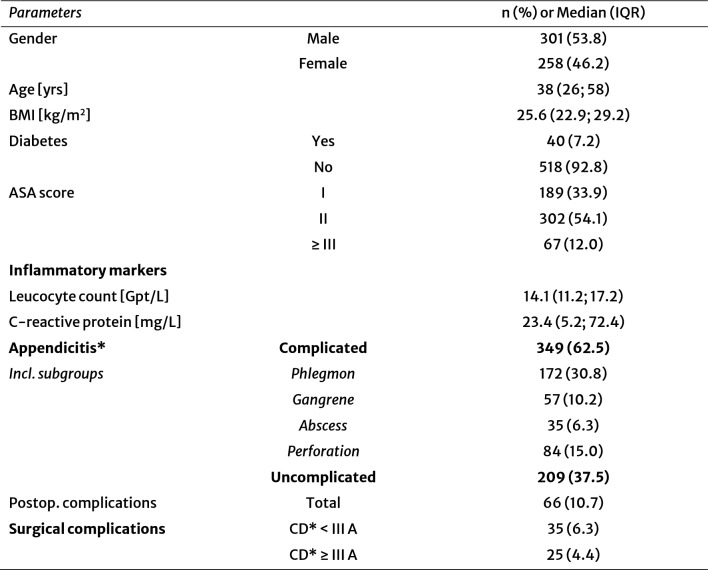
Patient characteristics

*BMI* body mass index, *ASA* American Society of Anaesthesiology, *CD* Clavien–Dindo classification

### CRP level as biomarker for the severity of acute appendicitis

**Fig. 1:**
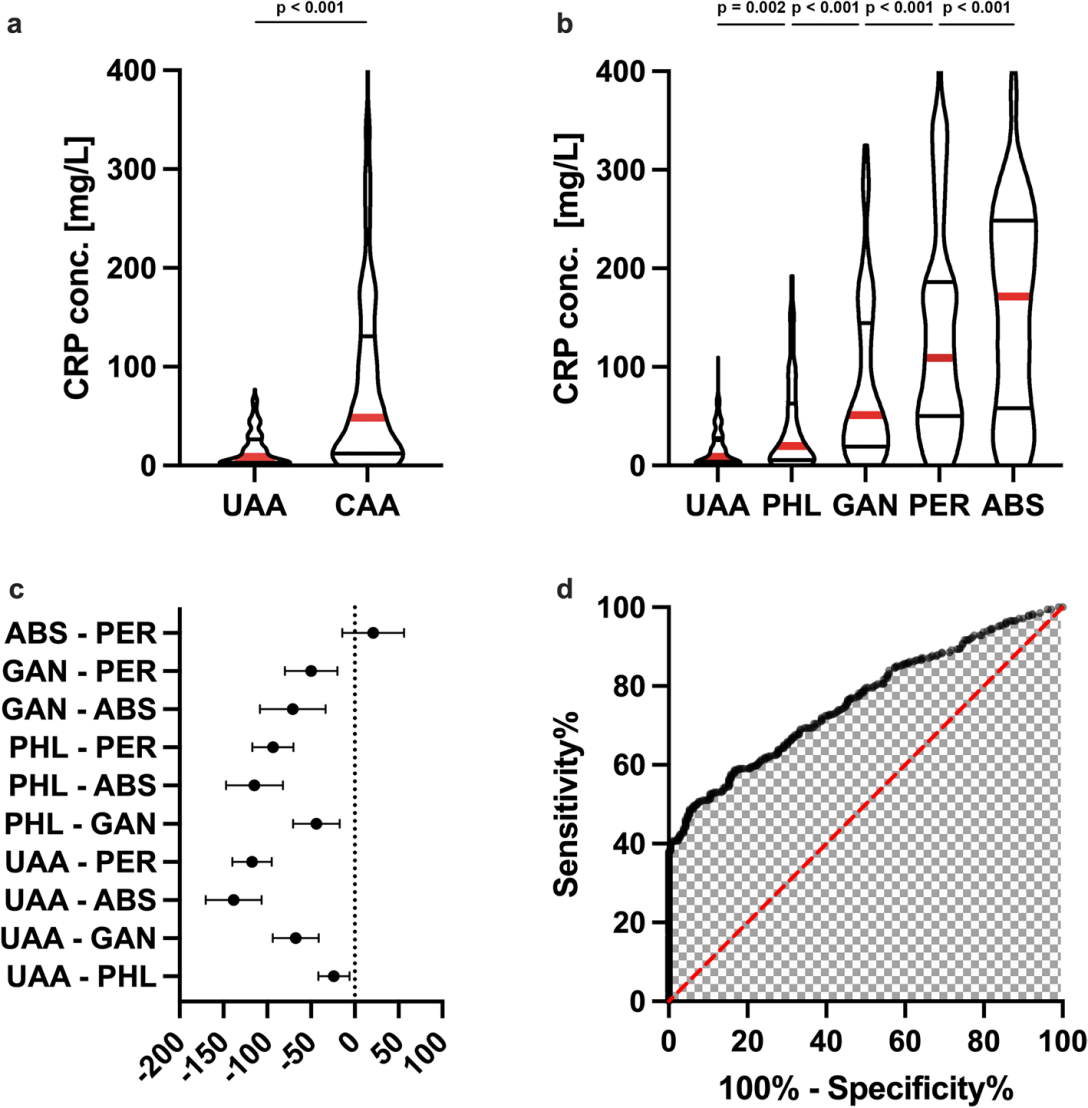
Preoperative CRP concentrations of patients with uncomplicated (UAA) and complicated (CAA) acute appendicitis. **a** Comparison of patients with uncomplicated- and complicated acute appendicitis, including cases of phlegmonous appendicitis according to EAES guidelines (*p* < 0.001). **b** Subgroup analysis of acute appendicitis revealed a progressive increase in preoperative CRP concentration in correlation with disease severity compared to UAA (Kruskal–Wallis test, *p* < 0.001). **c** A forest plot illustrating the effect size of disease severity on CRP concentration. The dots represent the median values, while the horizontal lines indicate the 95% confidence intervals. A value of 0 represents no effect in CRP concentration. **d** Receiver operating characteristic (ROC) analysis of preoperative CRP concentration demonstrated a good predictive value for distinguishing CAA (AUC: 0.76; CI: 0.72–0.80). The red dashed line represents an AUC of 0.5, indicating no predictive ability

CRP levels were significantly higher in complicated compared to uncomplicated appendicitis (Median 48.4 vs. 8.8 mg/L, p < 0.001; Fig. 1a). Subgroup analysis showed progressively increasing CRP levels with disease severity, with the highest values observed in abscess-forming and perforated cases (Fig. 1b–c). The diagnostic performance of CRP in distinguishing CAA from UAA was good (AUC 0.76, 95% CI: 0.72–0.80), with an optimal cutoff of 52.65 mg/L (sensitivity 49%, specificity 95%) (Fig. 1d).

### Leucocyte counts and their prognostic value in appendicitis classification

**Fig. 2:**
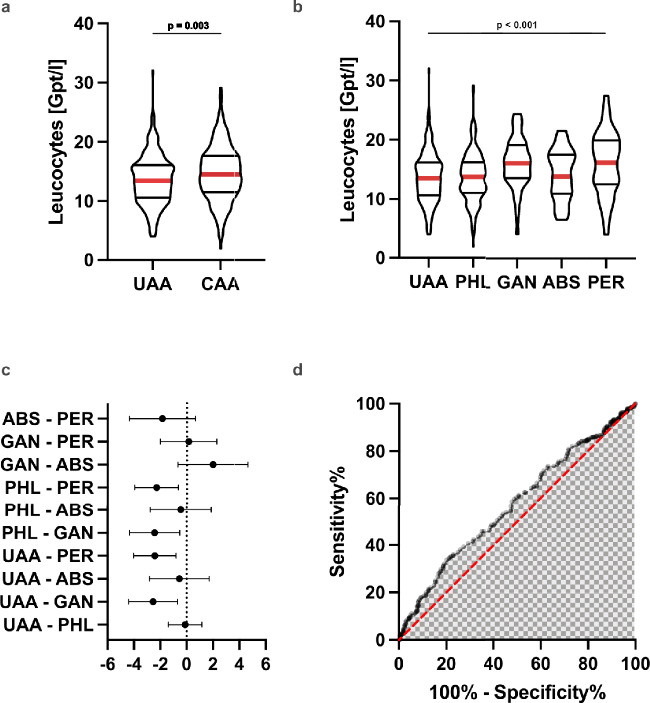
Preoperative Leucocyte counts of patients with uncomplicated (UAA) and complicated (CAA) acute appendicitis. **a** Comparison of preoperative Leucocyte counts of patients with acute appendicitis **b** The subgroup analysis using one-way ANOVA reveals no difference between UAA and phlegmonous appendicitis. However, leucocyte counts were higher in patients with gangrenous- and perforated acute appendicitis compared to UAA. **c** Forest plot of posthoc analysis utilizing Bonferroni multiple comparison test illustrates that leucocyte counts differ only between phlegmonous- (PHL) and gangrenous appendicitis (GAN), as well as phlegmonous- (PHL) and perforated appendicitis (PER). There was no other significant difference of leucocytes between subgroups of CAA. A **d** ROC analysis demonstrates only poor diagnostic performance of leucocyte count for the presence of CAA

Leukocyte counts were slightly higher in patients with complicated vs. uncomplicated appendicitis (Median 14.5 vs. 13.4 Gpt/L, *p* = 0.003), but subgroup differences were modest. Diagnostic performance was poor (AUC 0.57, 95% CI: 0.53–0.62), with an optimal cutoff of 16.65 Gpt/L (sensitivity 34%, specificity 80%) (Fig. 2).

### CRP and leucocyte count and the risk of postoperative complications in acute appendicitis

**Fig. 3:**
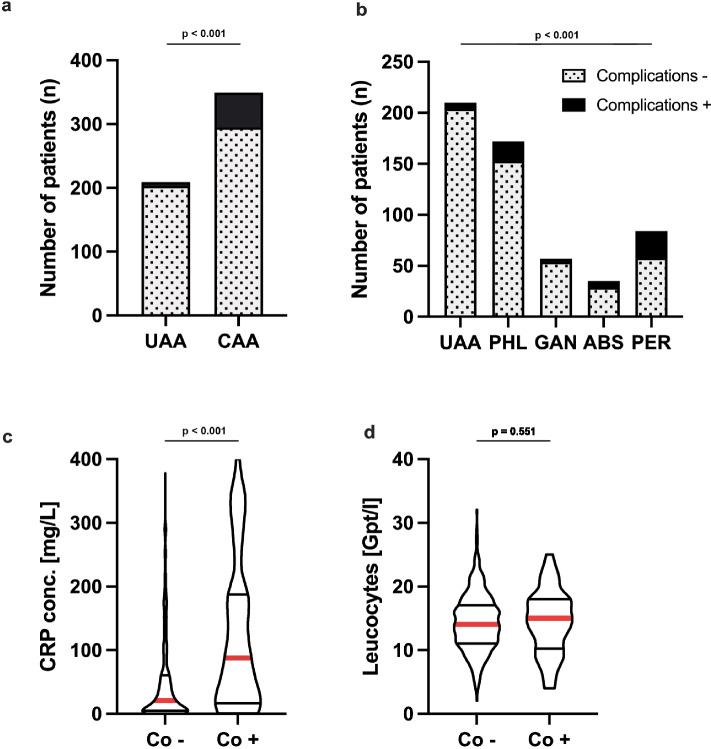
The influence of CRP levels and leucocyte counts on the risk of postoperative complications. **a** The proportion of postoperative complications in complicated acute appendicitis (CAA) (*p* < 0.001) is higher than in UAA. **b** Also, the proportion of postoperative complications varies between subgroups of CAA (*p* < 0.001). **c** Patients with postoperative complications (Co +) had elevated CRP concentrations preoperatively compared to patients without development of complications (Co-). **d** However, preoperative leucocyte counts did not differ between patients with complications and patients without complications

A total of 558 patients were included in the analysis. 54 of 349 (15.47%) patients had postoperative complications (30d-morbidity) after complicated acute appendicitis (CAA), whereas only 6 of 209 (2.87%) patients suffered complications after surgery for uncomplicated acute appendicitis (UAA) (*χ*^*2*^ = 21.63, *p* < 0.001, Fig. 3a). Figure 3b illustrates the proportions of postoperative complications across appendicitis subtypes. 19 of 172 (11.0%) patients with phlegmonous appendicitis (PHL) and 3 of 54 (5.28%) patients with gangrenous appendicitis developed postoperative complications. 6 of 35 (17.14%) with abscesses and 26 of 84 (30.95%) patients with perforated appendicitis had postoperative complications.

The odds ratio (OR) was 6.19 (95% CI: 2.64–13.55), indicating that patients with CAA had 6.2-fold higher odds of experiencing postoperative complications compared to those with UAA (not shown). The logistic regression model demonstrated that the severity of appendicitis according to EAES guidelines influenced postoperative complications (LR χ^2^(4) = 55.08, *p* < 0.001, Table 2). Patients with perforated appendicitis had the highest estimated odds for complications (aOR: 8.1, 95% CI: 4.2–15.2, *p* < 0.001). Abscess-forming appendicitis was also associated with an increased odds for complications (aOR: 5.7, 95% CI: 2.8–11.4, *p* = 0.001)**.** Gangrenous appendicitis (aOR: 4.5, 95% CI: 2.1–9.5; *p* = 0.002) and phlegmonous appendicitis (aOR: 2.3, 95% CI: 1.2–4.3, *p* = 0.015) were linked to a moderate but relevant increase in complications. Also, there was a difference in CRP levels between patients with and without postoperative complications (z = − 5.633, *p* < 0.001; Fig. 3c), indicating that elevated CRP levels were associated with a higher risk of complications. Patients who developed postoperative complications had a median CRP level of 96.6 mg/L (IQR: 19.8–189.6) compared to 22.14 mg/L (IQR: 4.8–63.2) in those without complications. In contrast, leucocyte counts did not relevantly differ between the groups (*z* = − 0.596, *p* = 0.5513) (Fig. 3d).

**Table 2:**
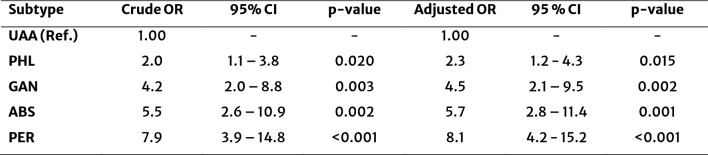
Risk of postoperative complications by appendicitis subtype

*Ref* Reference, *UAA* Uncomplicated acute appendicitis, *PHL* Phlegmonous acute appendicitis, *GAN* Gangrenous acute appendicitis *PER* Perforated acute appendicitis, *ABS* Abscess, *OR* Odds ratio, *CI* Confidence interval

All subtypes of complicated acute appendicitis (CAA) showed increased odds for development of postoperative complications compared to uncomplicated acute appendicitis (UAA). Phlegmonous appendicitis (PHL) classified as CAA according to EAES guidelines had a 2.3-fold increase of odds compared to UAA to develop postoperative complications.

### Association between CRP and leucocyte count and length of hospital stay

**Fig. 4:**
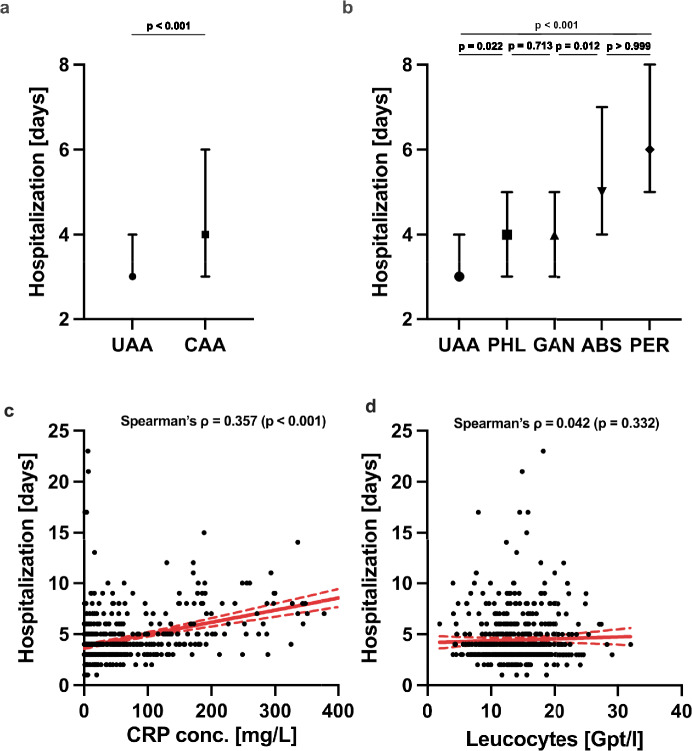
Association between CRP levels, leucocyte count, and length of hospital stay in patients with uncomplicated (UAA) and complicated appendicitis (CAA). **a** The length of stay in hospital is increased in complicated acute appendicitis (CAA) compared to uncomplicated acute appendicitis (*p* < 0.001). **b** Subgroup analysis with the results of Kruskal–Willis test and Dunn’s posthoc analysis with multiple comparisons. **c** Spearman’s correlation analysis reveals only moderate correlation between CRP concentration and hospitalization. **d** Preoperative leucocyte counts do not correlate with length of stay (*p* = 0.332)

We compared the length of hospital stay between patients with uncomplicated (UAA) and complicated appendicitis (CAA) (Fig. 4a). The analysis revealed a difference between the two groups (Z = − 8.239, *p* < 0.001). Patients with CAA had a longer median hospital stay (Median = 4 days, IQR = 3–6) compared to those with UAA (Median: 3 days, IQR: 3–4). Figure 4b shows the subgroup analysis with differences of hospital stay between UAA (Median = 3; IQR: 3–4) and phlegmonous- (Median = 4; IQR: 3–5; *p* = 0.022), gangrenous- (p < 0.001), and perforated appendicitis (Median = 6; IQR: 5–8; *p* < 0.001), as well as abscesses (Median = 5; IQR: 4–7; *p* < 0.001), respectively. Post hoc analysis showed longer length of hospital stay for perforated appendicitis compared to other subgroups (all *p* < 0.001), except for abscesses (*p* > 0.999). Phlegmonous- and gangrenous appendicitis also did not show a difference in length of stay (*p* > 0.713).

To test the association between intraoperative classification of appendicitis according to EAES guidelines on length of hospital stay, we performed a negative binomial regression model (Table 3), which revealed an association between the intraoperative classification of appendicitis and the length of hospital stay (LR χ^2^(4) = 138.03, *p* < 0.001). The analysis demonstrated that patients with CAA had longer hospitalizations compared to those with UAA. In this model, patients with perforated appendicitis (PER) had the longest estimated hospital stay (*β* = 0.64, *p* < 0.001), corresponding to an incidence rate ratio (IRR) of 1.89 (95% CI: 1.68–2.12), indicating a 89% increase (almost twofold) in hospital length of stay compared to the reference of UAA. Similarly, abscess-forming appendicitis (ABS) was associated with a 64% longer hospital stay (*β* = 0.49, *p* < 0.001; *IRR* = 1.64, 95% CI: 1.39–1.91). Gangrenous appendicitis (GAN) was linked to a 30% increase in hospitalization (*β* = 0.26, *p* = 0.001; *IRR* = 1.30, 95% CI: 1.12–1.47), while phlegmonous appendicitis (PHL) resulted in a 21% longer hospital stay (*β* = 0.19, *p* < 0.001; *IRR* = 1.21, 95% CI: 1.09–1.35).

**Table 3:**
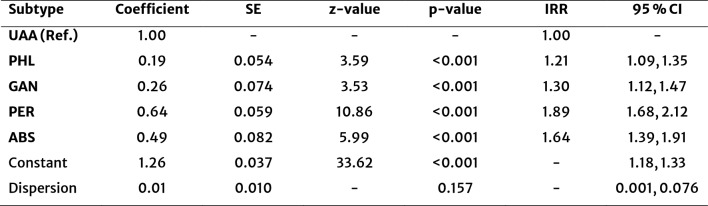
Adjusted incidence rate ratios (IRRs) for length of hospital stay by appendicitis subtype

*UAA* Uncomplicated acute appendicitis, *PHL* Phlegmonous AA, *GAN *Gangrenous AA, *PER* Perforated AA, *ABS *Abscess, *SE* Standard error, *IRR* Incidence rate ratio, *CI* Confidence interval

Next, we investigated the dependency of length of hospital stay on preoperative CRP concentration and leucocyte counts utilizing Spearman’s correlation analysis (Fig. 4c, d). CRP levels moderately correlated with hospital stay (*r* = 0.36, *p* < 0.001), whereas leukocyte count showed no relevant association (*p* = 0.332).

## Discussion

Acute appendicitis remains one of the most common surgical emergencies with a high incidence in western countries. While appendectomy is still considered the gold standard, non-operative treatment (NOT) with antibiotics has gained interest for selected cases [11].

The distinction between uncomplicated (UAA) and complicated appendicitis (CAA) plays a central role in treatment decisions [10, 11, 31]. By categorizing phlegmonous appendicitis (PHL) as a complicated form, the European Association for Endoscopic Surgery (EAES) set a new standard in the classification of acute appendicitis in 2015 [15]. This concept was accepted and introduced in the first German guideline for treatment of acute appendicitis in 2020 [17].

According to EAES guidelines, UAA is defined as inflammation of the appendix without surrounding tissue reaction or perforation [15]. Conversely, CAA includes cases with free peritoneal fluid, phlegmonous tissue reaction, abscess formation, gangrene, or perforation [15] Still the classification of PHL has been inconsistent across studies, which limits comparability and affects clinical decision-making until now. Therefore, our study aimed to assess the relevance of counting PHL as CAA based on inflammatory markers, postoperative complications, and hospital stay [32].

C-reactive protein (CRP) and leucocyte count are established markers in the diagnosis of acute appendicitis [21]. Previous studies suggest that CRP has higher diagnostic value, particularly in advanced stages of AA [33]. However, comparisons across studies are limited by inconsistent classification systems [21]. In particular, the varying categorization of phlegmonous appendicitis as either UAA or CAA affects the interpretation of inflammatory markers. To address this, we analyzed CRP and leucocyte counts based on the EAES 2015 criteria, focusing on phlegmonous cases as part of the complicated group. Our data show that CRP levels increase with disease severity and are higher in all complicated subtypes compared to UAA. This supports the use of CRP as a marker of disease severity and aligns with the classification of phlegmonous appendicitis as a complicated form. Despite CRP showed only moderate diagnostic accuracy, it is more reliable than leucocyte count. Leucocyte count failed to reliably distinguish between appendicitis subgroups. These results are consistent with previous findings and confirm the limited diagnostic utility of leucocytes in clinical decision-making [34].

We observed that phlegmonous appendicitis, when classified as CAA according to EAES guidelines, was associated with an increased risk of postoperative complications (2.3-fold increase) compared to UAA. Among all subtypes, perforated and abscess-forming appendicitis had the highest complication rates, which emphasizes the need for timely surgery. Elevated CRP levels were also associated with a higher complication risk and may serve as an additional prognostic marker. In contrast, leucocyte count showed no significant association with postoperative outcomes.

Also, stages of acute appendicitis correlated with hospital stay. Patients with CAA were longer hospitalized compared to UAA, with perforated and abscess-forming appendicitis showing the longest durations. Phlegmonous appendicitis also resulted in a 21% increase in length of stay. CRP levels moderately correlated with hospital stay, supporting their limited use for anticipation the need of hospitalization. In contrast, leucocyte count did not show this association.

While the single-center design may limit generalizability, the inclusion of a large and diverse patient cohort enhances the external validity of our findings. Additional limitations include the retrospective study design, which may be prone to documentation bias and missing data. Furthermore, CRP and leucocyte measurements were only taken at a single time point, which may not reflect dynamic inflammatory changes. Lastly, no long-term follow-up beyond the 30-day postoperative period was conducted, which may underestimate delayed complications or readmissions.

Our findings have implications for both clinical decision-making and future research. In clinical practice, the results support the EAES 2015 classification system by showing that phlegmonous appendicitis is still associated with increased morbidity and prolonged hospitalization. This reinforces the rationale for early surgical intervention in phlegmonous cases rather than conservative management. Moreover, CRP emerges as a useful, readily available biomarker to assist in the preoperative stratification of disease severity, potentially guiding decisions on timing and urgency of surgery. From a research perspective, these data highlight the need for standardized definitions and classifications in appendicitis research and suggest that future trials on NOT should clearly distinguish between uncomplicated and phlegmonous cases using objective markers such as CRP.

## Conclusion

Overall, phlegmonous appendicitis is associated with elevated CRP, higher complication rates, and prolonged hospitalization, which supports the EAES classification of 2015. Applying CRP-based thresholds may assist in preoperative stratification and guide decisions between surgical and conservative management. Leucocyte count, in contrast, is of limited diagnostic and prognostic utility. Further research is needed to explore the impact of standardized classification of patients with PHL into CAA, as well as the effectiveness of NOT in patients with UAA.

## Data Availability

The datasets generated and/or analyzed during the current study are available from the corresponding author on reasonable request.
